# Genome Sequence of the Proteorhodopsin-Containing Bacterium Flavobacterium sp. Strain TH167, Isolated from Cyanobacterial Aggregates in a Eutrophic Lake

**DOI:** 10.1128/genomeA.00217-18

**Published:** 2018-04-05

**Authors:** Haiyuan Cai, Feng Chen

**Affiliations:** a State Key Laboratory of Lake Science and Environment, Nanjing Institute of Geography and Limnology, Chinese Academy of Sciences, Nanjing, China; b The Institute of Marine and Environmental Technology, University of Maryland Center for Environmental Science, Baltimore, Maryland, USA

## Abstract

Flavobacterium is the most abundant group of bacteria within the cyanobacterial aggregates in Lake Taihu, China. Here, we present the genome sequence and annotation of Flavobacterium sp. strain TH167. Genome analysis revealed the presence of a proteorhodopsin-encoding sequence, together with its retinal-producing pathway, indicating a putative photoheterotrophic lifestyle that generates energy from light.

## GENOME ANNOUNCEMENT

Lake Taihu is the third largest freshwater lake in China, located in the rapidly developing economically important Changjiang (Yangtze) River Delta. Microcystis spp. often form large mucilaginous blooms in the lake due to anthropogenic nutrient overenrichment. These bloom aggregates are composed of extracellular polymeric substances, mainly consisting of polysaccharides, proteins, lipids, and humic substances (1). It is known that many heterotrophic bacteria live in association with cyanobacteria (2, 3). Flavobacterium is the most abundant group of bacteria within the aggregates (3). Marine flavobacteria are a well-known reservoir of microbial rhodopsins (4, 5); however, proteorhodopsin (PR)-containing bacteria within cyanobacterial aggregates have not yet been studied. Here, we report the genome sequence of Flavobacterium sp. strain TH167, which was isolated from cyanobacterial aggregates during bloom in Lake Taihu, China.

Strain TH167 was isolated from cyanobacterial aggregates collected in the Meiliang Bay (31°30′N, 120°11′E) in Lake Taihu, China. The 16S rRNA sequence analysis revealed that strain TH167 belongs to the genus Flavobacterium.

Strain TH167 was cultivated in R2A broth at 28°C for 2 days. Genomic DNA was isolated using the Gentra Puregene Yeast/Bact. kit (Qiagen). Preparation of a paired-end sequencing library with the Nextera XT library preparation kit and sequencing of the library using the HiSeq PE150 system were performed as described by the manufacturer (Illumina, San Diego, CA, USA). *De novo* assembly of all trimmed reads with SOAP*denovo* version 2.0 (6) resulted in 228 contigs. Open reading frames were predicted using the NCBI Prokaryotic Genomes Annotation Pipeline version 4.2 and the Rapid Annotations using Subsystems Technology (RAST) server (7). The genome of Flavobacterium sp. TH167 consists of 3.24 Mbp, with 40.4% GC content. The genome contained 2,954 protein-coding sequences, 3 rRNAs (5S, 16S, and 23S), and 38 tRNAs.

The genome of strain TH167 contained a full-length PR gene. The PR-encoding sequence codes for a green- and blue-light-absorbing PRopsin (8) of 243 amino acid residues. It shows sequence features suggestive of proton pump activity from the inside to the outside of the bacterial cell, leading to a proton motive force across the cell membrane (8, 9). A *blh* gene encoding a 15,15′-β-carotene dioxygenase, whose gene product is predicted to produce two molecules of retinal from β-carotene, was present immediately flanking the PR gene. The reading frames of the PR and *blh* genes were oriented in opposite directions, similar to other PR-containing *Bacteroidetes* (9). In addition, all genes required for β-carotene biosynthesis, including those for phytoene dehydrogenase (*crtI*), phytoene synthase (*crtB*), and β-carotene hydroxylase (*crtZ*), which located together in a cluster and under transcriptional control by a putative regulator (MerR family), geranylgeranyl diphosphate synthase (*crtE*), and lycopene β-cyclase (*crtY*), were located in the genome of strain TH167. In addition, cyanophycin granules were present in the cells, as indicated by the transmission electron microscopy study and genes coding for cyanophycin synthase in the genome of the strain. The genome of strain TH167 should provide further insight into the physiological and ecological functions of Flavobacterium spp. dominating within cyanobacterial aggregates.

### Accession number(s).

This whole-genome shotgun project has been deposited in DDBJ/ENA/GenBank under the accession no. NOXX00000000.
